# DPSSD: Dual-Path Single-Shot Detector

**DOI:** 10.3390/s22124616

**Published:** 2022-06-18

**Authors:** Dongri Shan, Yalu Xu, Peng Zhang, Xiaofang Wang, Dongmei He, Chenglong Zhang, Maohui Zhou, Guoqi Yu

**Affiliations:** 1 School of Mechanical Engineering, Qilu University of Technology (Shandong Academy of Sciences), Jinan 250300, China; 1043119034@stu.qlu.edu.cn (Y.X.); 1043119003@stu.qlu.edu.cn (C.Z.); 1043119049@stu.qlu.edu.cn (M.Z.); 1043119037@stu.qlu.edu.cn (G.Y.); 2 School of Information and Automation Engineering, Qilu University of Technology (Shandong Academy of Sciences), Jinan 250300, China; zp@qlu.edu.cn (P.Z.); wxf2012@stu.xjtu.edu.cn (X.W.); hedm@sdas.org (D.H.)

**Keywords:** convolution neural networks, object detection, single-stage, multi-scale

## Abstract

Object detection is one of the most important and challenging branches of computer vision. It has been widely used in people’s lives, such as for surveillance security and autonomous driving. We propose a novel dual-path multi-scale object detection paradigm in order to extract more abundant feature information for the object detection task and optimize the multi-scale object detection problem, and based on this, we design a single-stage general object detection algorithm called Dual-Path Single-Shot Detector (DPSSD). The dual path ensures that shallow features, i.e., residual path and concatenation path, can be more easily utilized to improve detection accuracy. Our improved dual-path network is more adaptable to multi-scale object detection tasks, and we combine it with the feature fusion module to generate a multi-scale feature learning paradigm called the “Dual-Path Feature Pyramid”. We trained the models on PASCAL VOC datasets and COCO datasets with 320 pixels and 512 pixels input, respectively, and performed inference experiments to validate the structures in the neural network. The experimental results show that our algorithm has an advantage over anchor-based single-stage object detection algorithms and achieves an advanced level in average accuracy. Researchers can replicate the reported results of this paper.

## 1. Introduction

After the success of deep convolution neural networks (DCNN) [1] in the field of image classification, the object detection algorithm also introduces deep-learning technology and has achieved significant progress [2,3]. These new algorithms based on deep learning are much better than the traditional algorithm because the feature of the manual design is replaced with the feature representation computed via convolution neural networks. However, multi-scale feature learning is a critical problem of the detection algorithms based on deep learning. To optimize this problem and improve the detection effect of the single-stage multi-scale detector based on the anchor box, we conducted a relevant literature search and experiments.

**Figure 1:**
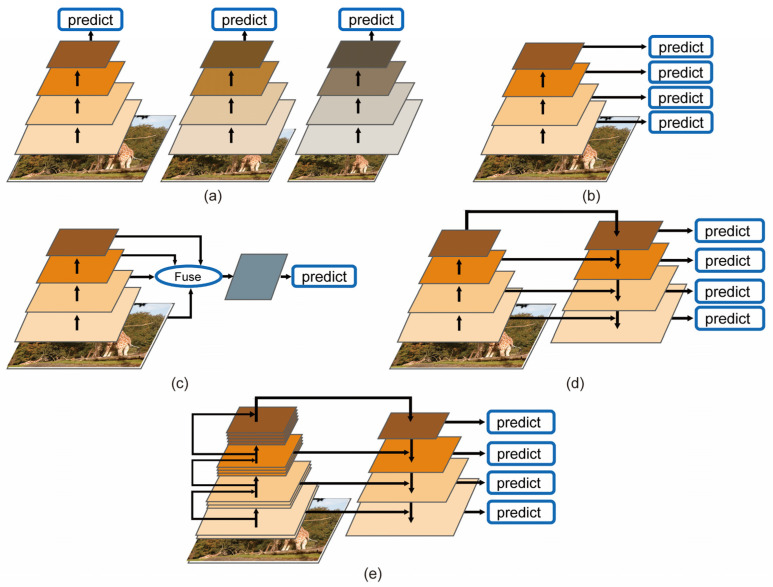
Four paradigms of multi-scale object detection. (**a**) Image Pyramid: It learns multiple detectors from different scale images. (**b**) Prediction Pyramid: It predicts on multiple feature maps. (**c**) Integrated Features: They predict on a single feature map generated from multiple features. (**d**) Feature Pyramid: It combines the structure of the prediction pyramid and integrated features. (**e**) Dual-Path Feature Pyramid: It uses the structure of the prediction pyramid and two methods of feature fusion.

In general, the objects are placed in a complex environment and have a large variance in scale; for example, in applications such as pedestrian detection, face detection and autonomous driving, the algorithm has to be robust to changes in the scale of the object [4]. It is critical to train a robust and discriminate feature to obtain good detection performance. There are four main paradigms to address the multi-scale feature learning problem: the image pyramid, the prediction pyramid, integrated features and the feature pyramid (Figure 1). SNIP [5] uses the image pyramid to solve the multi-scale problem, where each layer is responsible for a certain range of scales (Figure 1a). In this way, the same sample needs to be converted into different scales and repeatedly input to the network for training. This results in many redundant calculations. By fusing the shallow features rich in space and the deep features rich in semantics, the newly constructed features contain rich information and, thus, can detect the objects of different scales. Single-Shot MultiBox Detector (SSD) [6] and Multi-scale Deep Convolutional Neural Network (MSCNN) [7] use both shallow features rich in geometric information and deep features rich in semantic information to predict objects at different scales, which we call multi-scale prediction, using a prediction pyramid, where each layer is responsible for a certain scale of objects, as shown in Figure 1b. The Inside-Outside Network (ION) [8] and HyperNet [9] use integrated features to combine multiple layers of features to build a single feature map, and they make a final prediction based on it (Figure 1c). The Feature Pyramid Network (FPN) [10] uses the feature pyramid to integrate different scale features with lateral connections in a top-down fashion to build a set of scale-invariant feature maps to train multiple scale-dependent classifiers. This method also combines the deep semantic-rich features and the shallow spatially rich features (Figure 1d). FPN has significantly improved the performance of object detection algorithms and has achieved advanced state-of-the-art results in learning multi-scale features. However, these paradigms can only use information from a single layer of feature maps at different scales.

**Figure 2:**
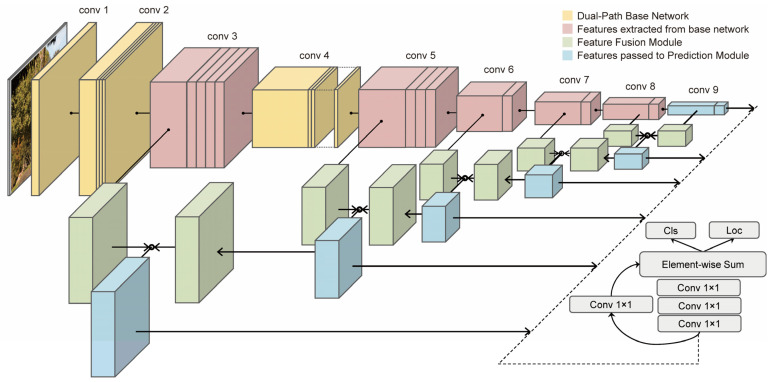
The architecture of the dual-path single shot detector. We designed a dual-path network and a feature fusion module to obtain six high-level and low-level features after fusion. Finally, the classification and bounding box regression were carried out by one-by-one convolution. The figure shows that several layers in the base network were extracted as the features for predicting objects of different sizes.

Feature fusion is the merging of different feature maps. In order to fully capture information at all levels of the feature map at different scales [4], we propose a paradigm to address multi-scale feature learning problems by using the dual-path feature pyramid (Figure 1e), which uses the structure of prediction pyramid and two methods of feature fusion, i.e., residual connection [1] and concatenation connection [11]. Figure 2 shows the overall structure of our detector. “Element-wise sum” denotes the matrix addition and is abbreviated as “Elts”. The overall framework consists of a base network, a feature fusion module and a prediction module. We used the idea of dual-path networks [12] to design our base network for the single-stage detector to obtain robust and discriminate features. Our dual-path network can generate six different resolutions of feature maps for multi-scale object detection. After experimental validation, we used a 3-by-3 convolution and deconvolution operation to fuse two feature maps adjacent to the resolution, and the fusion module contains five sub-networks to obtain five different scales of feature maps for prediction. The advantages of the single-stage object detection algorithm include a simple model training strategy and network structure and fast computing speed [4]. The whole detector retains the advantages of the single-stage algorithm and enables end-to-end training. Our object detector has the following innovations:

(a) We first introduced a dual-path network in the single-stage object detector by proposing a paradigm called the “Dual-Path Feature Pyramid”, as shown in Figure 1e. It combines two feature fusion methods, i.e., residual connection and concatenation connection.
(b) After experimental validation, a new feature fusion module was proposed to enhance the fusion of high-level semantic and low-level spatial features to further optimize the multi-scale feature learning problem.

## 2. Related Work

Currently, object detection can be divided into single stage and two stage. Two stage means that the object detection process is divided into a region proposal stage and a detection stage, while single stage means that both of these stages are carried out simultaneously. Object detection has many applications, such as face detection, pedestrian detection and automatic driving. However, multi-scale detection is the key to realize these applications [4].

The representative two-stage detectors include R-CNN [2], Fast-RCNN [13], Faster-RCNN [3] and R-FCN [14]. These algorithms first generate pooling and bounding box regression. The detection accuracy of two-stage detectors is good, but the frameworks limit the detection speed. Some researchers have been devoted to single-stage detectors, such as OverFeat [15], SSD [6] and YOLO [16]. The advantage of these detectors is that there is no need to generate region proposals, and each position on the input image may be the target object, using the end-to-end training method, so the detection speed is very fast. However, these methods have similar architectures for solving multi-scale detection problems. SNIP [5] and R-CNN [2] adopt the structure of Figure 1a in solving multi-scale problems. Fast-RCNN [13], Faster-RCNN [3], OverFeat [15], SSD [6], R-SSD [17] and R-FCN [14] use the structure of Figure 1b. The Inside-Outside Network (ION) [8], HyperNet [9] and STDN [18] use integrated features to combine multiple layers of features to build a single feature map, and they make a final prediction based on it (Figure 1c). DSSD [19] and FPN [10] use the feature pyramid paradigm to develop multi-scale detectors. Recent research advances, including M2Det [20], BPN [21] and ASFF [22], have proposed efficient feature fusion networks under the feature pyramid paradigm (Figure 1d).

However, current feature pyramid paradigms do not take full advantage of feature information at different scales when constructing feature pyramids, which limits the detection of multi-scale detectors [4]. To solve this problem, we propose a new feature pyramid structure, as shown in Figure 1e, which is mainly derived from the ideas of DSSD [19] and R-SSD [17]. Fu et al., proposed a Deconvolutional Single-Shot Detector (DSSD) [19], which adds a residual block to each feature map, then performs the element-wise product on the different scale feature maps for feature fusion. The advantage of DSSD is that it introduces a residual operation to scale the feature map. Jeong et al., proposed R-SSD [17], which uses rainbow concatenation through both pooling and deconvolution to improve the accuracy of the conventional SSD [6]. The advantage of R-SSD is that it uses a concatenation method to enrich the features of each level.

In sum, there are generally two main methods to further improve the detection accuracy of multi-scale objects. One is to use a residual block to build various feature pyramid structures, as in DSSD [19]. Another is the concatenation that uses the concatenation of multi-layer features to detect objects, as in R-SSD [17]. We combine these two approaches and propose the dual-path feature pyramid to optimize the multi-scale object detection problem, as shown in Figure 1e.

We used our improved dual-path network to extract the features of different resolutions, used the feature fusion module to fuse the different levels of features, and enhanced them through convolution and deconvolution operations. Our detector combines the advantages of two methods and, finally, obtains six robust and discriminate features to make a prediction.

## 3. Dual-Path Single-Shot Detector

In this section, the entire structure of the neural network is described, and the internal structure of each module is further detailed. In addition, the whole process of model training will be introduced in detail, including the construction of the programming environment, the setting of the training hyper-parameters and the loss function.

### 3.1. Convolution Neural Network

Our proposed network consists of three parts: a base network for feature extraction (Conv3, Conv5, Conv6, Conv7, Conv8 and Conv9); a feature fusion module for adjacent feature fusion; and a prediction module. We first use our dual-path network to extract features at six resolutions. The output of the last layer of the base network, Conv9, has the lowest resolution and is fed directly into the prediction module, which implements a linear combination of multiple channel features for classification and prediction by using one-by-one convolution operations and residual connections. Then, we input the Conv8 and Conv9 to the feature fusion module, and then input the obtained results and the features of the Conv7 to the feature fusion module, repeat the above process to obtain five fused feature maps with different resolutions, and finally pass them to the prediction module for the final object classification and localization.

#### 3.1.1. Dual-Path Network

The improved dual-path network combines the core ideas of ResNet [1], ResNeXt [23] and DenseNet [11]. The first stage contains a 7-by-7 convolution layer and a maximum pooling layer, and the remaining eight stages have similar structures. The first layer of each stage can be divided according to the channel dimension of the residual connection and the concatenation connection in the features and can choose whether to perform the down sampling operation or not. The later layers of each stage can increase the number of feature channels, deepen the number of network layers and improve the learning of the network capability. To retain as much sub-layer information as possible and ensure that we obtain the features at different scales [12], we skip Conv1, Conv2 and Conv4 and select the outputs of the Conv3, Conv5, Conv6, Conv7, Conv8 and Conv9 as the original feature maps, whose structure is shown in Table 1.

**Figure 3:**
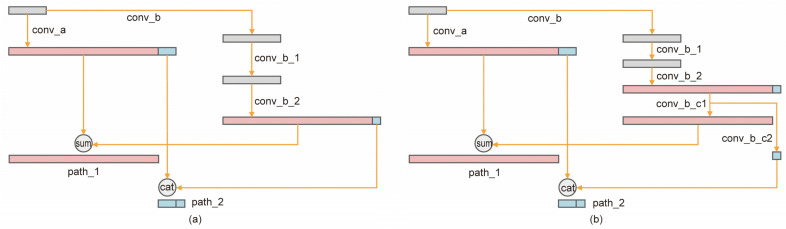
Two paradigms of dual-path block: (**a**) Feature segmentation is realized by channel merging. (**b**) The 1 × 1 convolution operation is used for feature segmentation.

In the remaining layers of each stage, the two groups of features output from the first layer are channel merged first and then input to the next layer; the output of the next layer can again be divided into two groups by one-by-one or channel separation, and the two output features of the first layer corresponding to the channels are summed and channel merged, respectively, as shown in Figure 3. Figure 3 shows the specific implementation of each layer in Table 1, which is the structure of the dual-pathway base network and corresponds to the top row in Figure 2. Feature segmentation refers to the method of cutting existing features in the dimension of the channel to obtain multiple sets of features. We studied the ablation of these two feature segmentation paradigms.

#### 3.1.2. Feature Fusion Module

**Figure 4:**
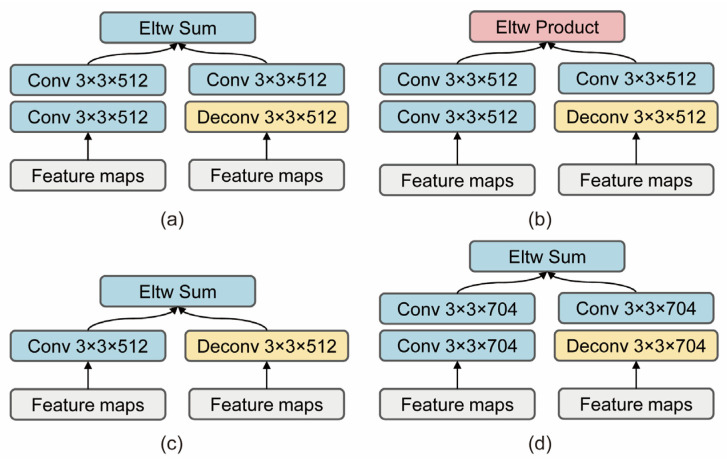
Four paradigms of feature fusion module. (**a**) Using a two-layer convolution operation, features are fused by sum. (**b**) Using a two-layer convolution operation, features are fused by the product. (**c**) Using a one-layer convolution operation, features are fused by sum. (**d**) Changing the number of channels for fusion features, and using a two-layer convolution operation, features are fused by sum.

**Figure 5:**
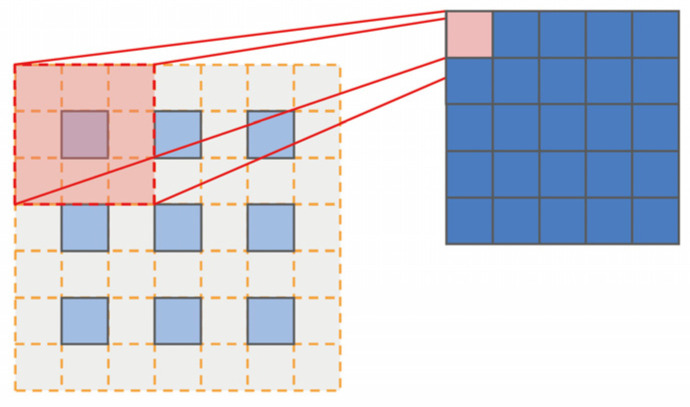
The paradigm of deconvolution operation.

The base network outputs feature maps of different resolutions that are responsible for predicting objects of different scales, and in the assignment principle, we continue the anchor frame matching principle in the SSD [6]. The DSSD [19] and RetinaNet [24] have demonstrated that, for single-stage object detection algorithms, the fusion of features at different levels can improve the detection effect. Therefore, we combine our own base network and experimental tests to design an efficient feature fusion module, which accepts two input feature maps to generate a fused feature. A 3-by-3 convolution operation is performed for the features with larger resolution in the input features, and conversely, a 3-by-3 deconvolution operation is performed for the smaller features; finally, the two results are summed to obtain the fused feature map, as shown in Figure 4. The effect of deconvolution is similar to bi-linear interpolation, which can improve the resolution of the feature map. The following describes the specific implementation process of the deconvolution kernel, as shown in Figure 5.

The deconvolution operation can be considered the inverse operation of convolution in terms of resolution. As is well known, there is a mathematical relationship between the resolution of the input and output in the convolution layer, and the mathematical expression is as follows:

(1) $$O_{n}=\left[\frac{I_{n}+2P_{n}-K_{n}}{S_{n}}\right]+1\;\left(n=h,w\right)$$

where $O_{n}$ is the layer of output size, $I_{n}$ is the layer of input size, $P_{n}$ is the layer of padding size, $K_{n}$ is the operation kernel size, $S_{n}$ is the convolution stride size and n represents the two optional dimensions of height and width. The output channel depends on the number of convolution kernels.

As the inverse of convolution, the mathematical formula for the deconvolution is expressed as the following:

(2) $$O_{n}^{\prime }=\left(I_{n}^{\prime }-1\right)\times S_{n}+K_{n}-2P_{n}+m\;\left(n=h,w\right)$$

where m is the layer of output padding, and it ranges from 0 to $S_{n}-1$. Due to the rounding operation, one input will correspond to m outputs when $S_{n}$ is greater than 1. The deconvolution seems to be the inverse process of convolution; however, there is no reversible relationship between the two in terms of numerical computation, except for feature resolution. The deconvolution layer is just an ordinary convolution layer, which is also needed in order to learn by gradient descent in a neural network. Therefore, for each deconvolution layer, we can actually use another convolution layer to perform the recovery as well.

The feature fusion module can fuse features of different channels at different levels. We set up ablation experiments with the number of fusion channels, fusion method and fusion layers, as shown in Figure 4.

#### 3.1.3. Prediction Module

**Figure 6:**
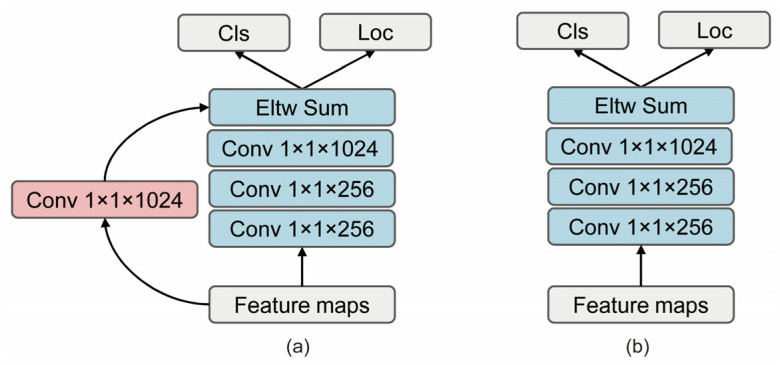
Two paradigms of prediction module: (**a**) prediction module with a residual connection; (**b**) prediction module without residual connection.

The prediction module has two sub-networks, one for classification prediction and the other for localization prediction, operating independently on each feature map. To ensure the reuse of the prediction results for the lower-level features, we designed a jump connection to connect the first layer and the sum of the final output layer features, as shown in Figure 6, and the experimental results are shown in Table 2.

### 3.2. Training Model

Our detector is developed using the PyTorch [25] framework. It is trained on NVIDIA TITAN Xp GPU. Our training strategy is almost the same as SSD [6], including a data augmentation trick following SSD [6], e.g., random flip, random scale, random crop, random brightness and random rotation, and an SGD (Stochastic Gradient Descent) solver. We perform pre-training on the Imagenet+5k, which means that the network has been pre-trained on Imagenet5k before being fine-tuned on Imagenet1k, and then further trained on the PASCAL VOC datasets and COCO datasets using the strategy of batch size of 14, learning rate of 0.001 and 120,000 iterations, with a 10-fold learning rate reduction at the 80,000th and 100,000th batches of the training process, and obtain two models with input image resolutions of 320 and 521.

In training the model with an input image of 320 pixels, the six feature map anchor boxes have step parameters of 8, 16, 32, 64, 107 and 320; minimum size parameters of 21, 45, 99, 153, 107 and 320; maximum size parameters of 45, 99, 153, 207, 261 and 315; and feature map resolution parameters of 1, 3, 5, 10, 20 and 40. For experiments on the PASCAL VOC datasets, anchor box aspect ratios of 1.6, 2.0 and 3.0 were used to generate eight anchor boxes per anchor point, and for experiments on the COCO datasets, anchor box aspect ratios of 2:3 were used to generate six anchor boxes per anchor point for comparison with the relevant models and following DSSD [19].

The training loss function consists of the combination of the localization loss Smooth L1 and the classification loss Softmax. The offset encoding of the ground truth of object localization is required before training, which can effectively reduce the learning difficulty, and the mathematical formula is as follows:

(3) $$L\left(X,c,l,a,g\right)=\frac{1}{N}\left(L_{conf}\left(X,c\right)+\alpha L_{loc}\left(X,l,a,g\right)\right)$$

(4) $$L_{conf}\left(X,c\right)=-\overset{N}{\underset{i\in Pos}{\sum }}X_{ij}^{p}\log\left(\hat{c_{i}^{p}}\right)-\underset{i\in Neg}{\sum }\log\left(\hat{c_{i}^{0}}\right).\;$$

(5) $$\mathrm{where}\;\hat{c_{i}^{p}}=\frac{\exp\left(c_{i}^{p}\right)}{\sum_{p}\exp\left(c_{i}^{p}\right)}\;\left(5\right)$$

(6) $$L_{loc}\left(l,a,g\right)=\overset{N}{\underset{i\in Pos}{\sum }}\underset{m\in \left\{cx,cy,w,h\right\}}{\sum }smooth_{L1}\left(l_{i}^{m}-\hat{g_{j}^{m}}\right).\;$$

(7) $$\hat{g_{j}^{cx}}=\left(g_{j}^{cx}-a_{i}^{cx}\right)/a_{i}^{w}.\;$$

(8) $$\hat{g_{j}^{cy}}=\left(g_{j}^{cy}-a_{i}^{cy}\right)/a_{i}^{h}.\;$$

(9) $$\hat{g_{j}^{w}}=\log\left(g_{j}^{w}/a_{i}^{w}\right)$$

(10) $$\hat{g_{j}^{h}}=\log\left(g_{j}^{h}/a_{i}^{h}\right)$$

(11) $${\mathrm{smooth}}_{L_{1}}\left(x\right)=\{\begin{array}{cc} 0.5x^{2} & \mathrm{if}\;\left|x\right|<1\\ \left|x\right|-0.5 & \mathrm{otherwise} \end{array}.\;$$

where X is the prediction vector for classification, α. is to balance the importance of the two losses, c is the classification label, l is the prediction vector for localization, a is the coordinate of the anchor box and g indicates the offset of the ground truth with respect to the anchor box. $L_{conf}$ is the classification loss, $L_{loc}$ is the localization loss, i is the index of anchor boxes, j is the index of the ground truth in an image and p is the index of each category in the classification vector. In Equations (7)–(10), x and y are the coordinates of the center point of the bounding box; w and h are the width and height of the bounding box.

Equations (7)–(11) are brought into Equation (6) to obtain the complete expression of the localization loss, Equation (5) is brought into Equation (4) to obtain the classification loss, and finally, Equations (4) and (6) are substituted into Equation (3) to obtain the total loss expression.

## 4. Experiments

### 4.1. Experiment Consideration

Our detector was evaluated on the PASCAL VOC [26] and COCO [27] datasets, the former with 20 object classes and the latter with 80 object classes. For the PASCAL VOC datasets, we followed the protocol in [10] and combined *VOC 2007 trainval* and *VOC 2012 trainval* as training sets for training and testing on the *VOC 2007 test*. For the COCO datasets, to compare with the previous algorithm, we combined *train2014* and *valminusminival2014* as training sets for training and testing on the *test-dev2015* test set.

We used the mean accuracy (mAP) as the core criterion for evaluation. For PASCAL VOC, we used an IOU (Intersection over Union) threshold of 0.5 to report the mAP score. For COCO, we used the evaluation matrix provided by the datasets itself. Experiments on PASCAL VOC and COCO are to verify the effectiveness of our proposed dual-path pyramid paradigm. Ablation experiments on the PASCAL VOC were used to explore different network structures of DPSSD.

The GPU we used was TITAN Xp/PCle/SSE2, and the CPU was Intel Core I7-8700K CPU @ 3.70ghz × 12. The training time of the model on PASCAL VOC datasets was 19 h and on the COCO datasets was 41 h.

### 4.2. Experiment on PASCAL VOC

We designed a dual-path network that generates feature maps with different depths and resolutions and enables the fusion of feature maps with different resolutions through a feature fusion module we designed specifically for it, which is a multi-scale object detection paradigm that learns more discriminate features.

We compared it with similar algorithms which are improved based on the SSD [6] to demonstrate that our proposed multi-scale feature learning paradigm has better detection results. SSD [6] belongs to (b) structure in Figure 1. STDN [18] belongs to (c) structure in Figure 1. DSSD [19] belongs to (d) structure in Figure 1. DPSSD (ours) belongs to (e) structure in Figure 1. The experimental results are shown in Table 3. The accuracy of DPSSD320 is 2.6% higher than that of DSSD321; DPSSD512 is 1.4% higher than DSSD513; DPSSD320 is 1.9% higher than STDN321; DPSSD512 is 2.0% higher than STDN513; DPSSD320 is 3.7% higher than that of SSD300; DPSSD512 is 3.4% higher than SSD512. Our model provides a significant improvement in terms of complexity and computational cost and enables the reuse of shallow features and the exploration of new features, which helps to generate a robust and discriminate feature with good detection performance.

**Figure 7:**
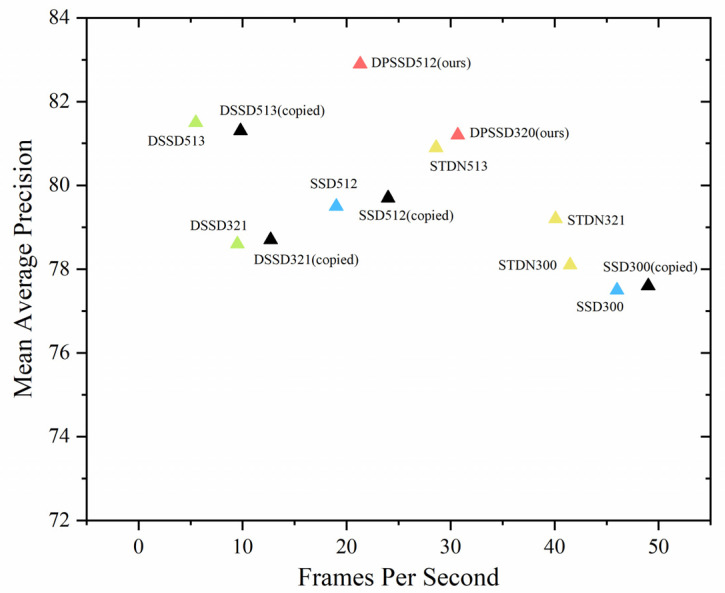
Accuracy and speed on PASCAL VOC2007.

These results reflect the improvement in the accuracy of our multi-scale detector and the effectiveness of our dual-path feature pyramid in object detection. We believe that the reason for this is that our designed dual-path network with the feature fusion module increases the amount of information in the feature pyramid at different scales of feature maps and enhances connection between low-level features and high-level features. It simultaneously maintains a good computational speed, as shown in Figure 7.

The datasets contain a total of 20 classes of objects, and our model achieves good detection accuracy for aeroplane, bird, boat, bottle, car, chair, cow, person, plant and sofa. The gap between our method and the methods listed in the table is no more than 1.5% in terms of multi-scale detection effectiveness on other categories. This further validates the generalization ability of the model for multi-scale detection.

### 4.3. Ablation Experiment on the PASCAL VOC

We designed a series of comparative experiments on PASCAL VOC2007 [26] to verify the effectiveness and rationality of each module in DPSSD. The results are shown in Table 2.

In Table 2, DPN denotes the dual-path network, FFM denotes the feature fusion module and PM denotes the prediction module. DPN + PM indicate that we used our dual-path network to extract the CNN features at different depths to perform object detection. DPN + FFM indicate that we tried to obtain multi-scale feature maps by using a feature fusion module. Using only the base network, the mAP of DPSSD320 was 78.9% and was 5.1% higher than that of SSD [6]. This proves that the base network is effective.

From the first to the fifth rows of Table 2, we can see that the four different feature fusion modules more or less improved the detection accuracy, and the feature fusion module with the best effect increased the accuracy from 78.9% to 81.2%. We believe that the reason for this is that humans need to consider the geometry and category properties of an object in recognizing its category and locating its position, while the fusion of deep semantic information features and shallow geometric spatial features of neural networks is exactly in line with our human localization and recognition of objects in spatial locations.

The second and sixth row of Table 2 show that a prediction module with a residual connection can slightly improve the object detection accuracy from 80.9% to 81.2%. The reason for this is that the shortcut increases the reuse of features.

The division method of channels in each stage of our designed base network contains both one-by-one convolution and channel segmentation. As shown in the second and seventh row of Table 2, the channel segmentation approach had better detection results than the one-by-one convolution approach.

### 4.4. Experiment on the Microsoft COCO

We also trained two models, DPSSD320 and DPSSD512, on the Microsoft COCO datasets [27] to further evaluate our detectors, and the results are shown in Table 4 We trained on the union of train2014 and valminusminival2014 and tested on test-dev2015. The different train datasets of the methods listed in Figure 4 do not affect the evaluation under the same test datasets because the different train datasets only affect the training stage. The evaluation indicators in the table were carried out on the same datasets, and the whole evaluation process was carried out on the official server; the real labels are not open to the public.

We focus on comparing the four methods of SSD [6], DSSD [19], STDN [18] and DPSSD (ours) because they are single-stage methods and are the same except for the structure of the feature pyramid. The average accuracy of SSD300, DSSD321, STDN300 and DPSSD320 (ours) on the test-dev2015 test set reached 25.1%, 28.0%, 28.0% and 30.6%, respectively. SSD512, DSSD513, STDN513 and DPSSD512 (ours) reached 28.8%, 33.2%, 31.8% and 33.9%, respectively. It can be seen that the average accuracy of our proposed model for multi-scale object detection holds an advantage. The experimental results further validate the effectiveness of our proposed dual-path feature pyramid paradigm. As shown in Figure 1e and Figure 3a, the dual-path convolution block can improve the efficiency of the feature pyramid in object detection.

However, it can be seen that our algorithm DPSSD513 was slightly lower than DSSD513 in terms of average accuracy and average recall for small and large objects. The density of the proposed area and the accuracy of the location will affect the recall rate [28]. We used 6 anchor boxes for training at each anchor point. The number of anchor boxes determines the density of the proposed area. The denser the proposed area, the fewer missed detections and the greater the recall rate of the model, but it will greatly increase the computational cost. In addition, considering the detection accuracy on objects of different scales, we find that the model achieves 51.2% detection accuracy for medium-scale objects and 20.6% and 64.3% for small and large objects, respectively. Through comparison, it can be concluded that the detection effect of the DPSSD in small objects has a lot of space to improve. Small objects have fewer features to use because they have a small area in the image. The semantic information of the environment should be used to improve the small-object detection effect [29]. Modeling the semantic relationship between environment and object through neural networks is our next research focus.

### 4.5. Experiment on Inference Speed

We tested 4952 images from the *PASCAL VOC2007 test* datasets on a Titan Xp and Intel Core i7-8700K CPU @7.70GHz device at a batch size of 1 to calculate the inference speed of our DPSSD model. The main factors affecting the detection speed include the complexity of the model, the calculation and the transmission speed of the hardware.

As shown in Table 5. For comparison, we replicated the official codes and training models of SSD [6] and DSSD [19] and conducted the test on the same hardware environment. We focused on comparing the four methods of SSD (copied), DSSD (copied), STDN [18], and DPSSD (ours) because they are the same except for the structure of the feature pyramid.

We plotted a scatter plot of accuracy and speed, as shown in Figure 7, to visualize the advantages and disadvantages of each algorithm. A good detector should gradually move closer to the top right corner of the graph. It can be seen that the DPSSD320 had a good trade-off between speed and accuracy, and the DPSSD512 is highly accurate but relatively slow. The dual-path feature pyramid is more effective than other pyramid structures in the field of object detection.

## 5. Conclusions

Our contribution is validating the effectiveness of a new feature pyramid paradigm, named the dual-path feature pyramid. This paradigm can give researchers a new way of constructing their own feature pyramid to optimize multi-scale problems. We improved a dual-path network and a feature fusion module specifically for the anchor-based object detection algorithm, which greatly improves the quality of features extracted by convolution neural networks with powerful learning capabilities. To verify its effectiveness, we trained the Dual-Path Single Shot Detector (DPSSD) on PASCAL VOC and COCO datasets, following SSD [6] strategy, and used it for comparing with detectors that have different pyramid paradigms. The extensive experiments above show that the dual-path single shot detector can achieve a good trade-off between speed and accuracy. At 30.7 FPS, DPSSD320 obtained 81.2 mAP on VOC 2007. At 21.3 FPS, DPSSD512 obtained 82.9 mAP. It can be seen that our detector still has some advantages over the comparable state-of-the-art detection algorithms.

Subsequently, we will continue to do relevant research on object detection. Specifically, we will work on the problem of sample imbalance, explore what kind of technology can further improve the detection effect of small objects and research applications of object detector in edge computing [29].

## Figures and Tables

**Table 1:** Dual-path network architecture.

Layers		Parameters		Output	Layers	Parameters			Output
		Size/Stride	Groups			Size/Stride		Groups	
Conv1_1	conv	7 × 7/2	1	80 × 80 × 64	Conv5_1	conv_a	1 × 1/1	1	20 × 20 × 1024 20 × 20 × 384
	maxpool	3 × 3/2	1			conv_b	1 × 1/1	1	
Conv2_1	conv_a	1 × 1/1	1	80 × 80 × 256 80 × 80 × 48		conv_b_1	3 × 3/1	32	
	conv_b	1 × 1/1	1			conv_b_2	1 × 1/1	1	
	conv_b_1	3 × 3/1	32		Conv5_2 - Conv5_3	conv_b	1 × 1/1	1	20 × 20 × 1024 20 × 20 × 512(+128)
	conv_b_2	1 × 1/1	1			conv_b_1	3 × 3/1	32	
Conv2_2 - Conv2_3	conv_b	1 × 1/1	1	80 × 80 × 256 80 × 80 × 80		conv_b_2	1 × 1/1	1	
	conv_b_1	3 × 3/1	32		Conv6_1	conv_a	1 × 1/2	1	10 × 10 × 1024 10 × 10 × 384
	conv_b_2	1 × 1/1	1			conv_b	1 × 1/1	1	
Conv3_1	conv_a	1 × 1/2	1	40 × 40 × 512 40 × 40 × 96		conv_b_1	3 × 3/2	32	
	conv_b	1 × 1/1	1			conv_b_2	1 × 1/1	1	
	conv_b_1	3 × 3/2	32		Conv7_1	conv_a	1 × 1/2	1	5 × 5 × 1024 5 × 5 × 384
	conv_b_2	1 × 1/1	1			conv_b	1 × 1/1	1	
Conv3_2 - Conv3_4	conv_b	1 × 1/1	1	40 × 40 × 512 40 × 40 × 128(+32)		conv_b_1	3 × 3/2	32	
	conv_b_1	3 × 3/1	32			conv_b_2	1 × 1/1	1	
	conv_b_2	1 × 1/1	1		Conv8_1	conv_a	1 × 1/2	1	3 × 3 × 1024 3 × 3 × 384
Conv4_1	conv_a	1 × 1/2	1	20 × 20 × 1024 20 × 20 × 72		conv_b	1 × 1/1	1	
	conv_b	1 × 1/1	1			conv_b_1	3 × 3/2	32	
	conv_b_1	3 × 3/2	32			conv_b_2	1 × 1/1	1	
	conv_b_2	1 × 1/1	1		Conv9_1	conv_a	1 × 1/2	1	1 × 1 × 1024 1 × 1 × 384
Conv4_2 - Conv4_20	conv_b	1 × 1/1	1	20 × 20 × 1024 20 × 20 × 96(+24)		conv_b	1 × 1/1	1	
	conv_b_1	3 × 3/1	32			conv_b_1	3 × 3/2	32	
	conv_b_2	1 × 1/1	1			conv_b_2	1 × 1/1	1	

**Table 2:** Ablation study on PASCAL VOC 2007 test set.

Method	mAP	Anchor Boxes	Input Resolution
DPN (a) + PM (a)	78.9	17,080	320 × 320
DPN (a) + FFM (a) + PM (a)	81.2	17,080	320 × 320
DPN (a) + FFM (b) + PM (a)	80.6	17,080	320 × 320
DPN (a) + FFM (c) + PM (a)	80.8	17,080	320 × 320
DPN (a) + FFM (d) + PM (a)	80.5	17,080	320 × 320
DPN (a) + FFM (a) + PM (b)	80.9	17,080	320 × 320
DPN (b) + FFM (a) + PM(a)	67.1	17,080	320 × 320

**Table 3:** PASCAL VOC2007 test detection results. All models were trained on the joint training set of VOC 2007 *trainval* and 2012 *trainval* and were tested on the VOC 2007 test *dataset*.

Method	SSD300 [6]	SSD512 [6]	STDN300 [18]	STDN321 [18]	STDN513 [18]	DSSD321 [19]	DSSD513 [19]	DPSSD320 (Ours)	DPSSD512 (Ours)
**Network**	VGG	VGG	DenseNet-169	DenseNet-169	DenseNet-169	Residual-101	Residual-101	DPN	DPN
**mAP**	77.5	79.5	78.1	79.3	80.9	78.6	81.5	81.2	82.9
**aero**	79.5	84.8	81.1	81.2	86.1	81.9	86.6	88.5	87.9
**bike**	83.9	85.1	86.9	88.3	89.3	84.9	86.2	87	88
**bird**	76	81.5	76.4	78.1	79.5	80.5	82.6	82.3	87.1
**boat**	69.6	73	69.2	72.2	74.3	68.4	74.9	76.2	79.9
**bottle**	50.5	57.8	52.4	54.3	61.9	53.9	62.5	56.5	66.3
**bus**	87	87.8	87.7	87.6	88.5	85.6	89	88.7	88.5
**car**	85.7	88.3	84.2	86.5	88.3	86.2	88.7	88.2	89
**cat**	88.1	87.4	88.3	88.8	89.4	88.9	88.8	88.4	88.4
**chair**	60.3	63.5	60.2	63.5	67.4	61.1	65.2	67.4	71.2
**cow**	81.5	85.4	81.3	83.2	86.5	83.5	87	84.6	87.3
**table**	77	73.2	77.6	79.4	79.5	78.7	78.7	77.3	79.2
**dog**	86.1	86.2	86.6	86.1	86.4	86.7	88.2	86.7	88
**horse**	87.5	86.7	88.9	89.3	89.2	88.7	89	89	89.1
**mbike**	83.9	83.9	87.8	88	88.5	86.7	87.5	87.8	87.3
**person**	79.4	82.5	76.8	77.3	79.3	79.7	83.7	80.9	85
**plant**	52.3	55.6	51.8	52.5	53	51.7	51.1	59.5	59
**sheep**	77.9	81.7	78.4	80.3	77.9	78	86.3	84.3	86.1
**sofa**	79.5	79	81.3	80.8	81.4	80.9	81.6	83.7	81.9
**train**	87.6	86.6	87.5	86.3	86.6	87.2	85.7	87	86.2
**tv**	76.8	80	77.8	82.1	85.5	79.4	83.7	80.6	82.8

**Table 4:** COCO test-dev2015 detection results.

Method	Data	Network	Avg. Precision, IoU:			Avg. Precision, Area:			Avg. Recall, #Dets:			Avg. Recall, Area:		
			0.5:0.95	0.5	0.75	S	M	L	1	10	100	S	M	L
SSD300 [6]	trainval35k	VGG	25.1	43.1	25.8	6.6	25.9	41.4	23.7	35.1	37.2	11.2	40.4	58.4
SSD512 [6]	trainval35k	VGG	28.8	48.5	30.3	10.9	31.8	43.5	26.1	39.5	42.0	16.5	46.6	60.8
DSSD321 [19]	trainval35k	Residual-101	28.0	46.1	29.2	7.4	28.1	47.6	25.5	37.1	39.4	12.7	42.0	62.6
DSSD513 [19]	trainval35k	Residual-101	33.2	53.3	35.2	13.0	35.4	51.1	28.9	43.5	46.2	21.8	49.1	66.4
STDN300 [18]	trainval	DenseNet-169	28.0	45.6	29.4	7.9	29.7	45.1	24.4	36.1	38.7	12.5	42.7	60.1
STDN513 [18]	trainval	DenseNet-169	31.8	51.0	33.6	14.4	36.1	43.4	27.0	40.1	41.9	18.3	48.3	57.2
DPSSD320 (ours)	trainval35k	DPN	30.6	50.2	32.2	10.3	32.0	47.6	26.8	39.5	41.5	16.1	44.9	62.6
DPSSD512 (ours)	trainval35k	DPN	33.9	53.8	36.3	14.5	37.5	48.7	28.7	43.4	45.7	20.6	51.2	64.3

**Table 5:** The speed and accuracy of the algorithm are summarized as follows. The training data are the combination of VOC2007 *trainval* and VOC2012 *trainval*.

Method	Base Network	mAP	Speed (*fps*)	Anchor Boxes	GPU	Input Resolution
SSD300 [6]	VGG16	77.5	46	8732	Titan X	300 × 300
SSD512 [6]	VGG16	79.5	19	24,564	Titan X	512 × 512
SSD300 (copied)	VGG16	77.6	49	8732	Titan Xp	300 × 300
SSD512 (copied)	VGG16	79.7	24	24,564	Titan Xp	512 × 512
DSSD321 [19]	Residual-101	78.6	9.5	17,080	Titan X	321 × 321
DSSD513 [19]	Residual-101	81.5	5.5	43,688	Titan X	513 × 513
DSSD321 (copied)	Residual-101	78.7	12.7	17,080	Titan Xp	321 × 321
DSSD513 (copied)	Residual-101	81.3	9.8	43,688	Titan Xp	513 × 513
STDN300 [18]	DenseNet-169	78.1	41.5	13,888	Titan Xp	300 × 300
STDN321 [18]	DenseNet-169	79.2	40.1	17,080	Titan Xp	321 × 321
STDN513 [18]	DenseNet-169	80.9	28.6	43,680	Titan Xp	513 × 513
DPSSD320 (ours)	DPN	81.2	30.7	17,080	Titan Xp	320 × 320
DPSSD512 (ours)	DPN	82.9	21.3	43,680	Titan Xp	512 × 512

## Data Availability

Researchers can replicate the reported results of this paper. Please refer to this link for a detailed explanation. https://github.com/Willie-Xu/DPSSD, 11 February 2022.

## References

[1] He K., Zhang X., Ren S., Sun J. Deep residual learning for image recognition. Proceedings of the IEEE Conference on Computer Vision and Pattern Recognition.

[2] Girshick R., Donahue J., Darrell T., Malik J. Rich Feature Hierarchies for Accurate Object Detection and Semantic Segmentation. Proceedings of the 2014 IEEE Conference on Computer Vision and Pattern Recognition.

[3] Ren S., He K., Girshick R., Sun J. (2017). Faster R-CNN: Towards Real-Time Object Detection with Region Proposal Networks. IEEE Trans. Pattern Anal. Mach. Intell..

[4] Wu X.W., Sahoo D., Hoi S.C.H. (2020). Recent advances in deep learning for object detection. Neurocomputing.

[5] Singh B., Davis L.S. An analysis of scale invariance in object detection snip. Proceedings of the IEEE Conference on Computer Vision and Pattern Recognition.

[6] Liu W., Anguelov D., Erhan D., Szegedy C., Reed S., Fu C.-Y., Berg A.C. Ssd: Single shot multibox detector. Proceedings of the European Conference on Computer Vision.

[7] Cai Z., Fan Q., Feris R.S., Vasconcelos N. A unified multi-scale deep convolutional neural network for fast object detection. Proceedings of the European Conference on Computer Vision.

[8] Bell S., Zitnick C.L., Bala K., Girshick R. Inside-outside net: Detecting objects in context with skip pooling and recurrent neural networks. Proceedings of the IEEE Conference on Computer Vision and Pattern Recognition.

[9] Kong T., Yao A., Chen Y., Sun F. Hypernet: Towards accurate region proposal generation and joint object detection. Proceedings of the IEEE Conference on Computer Vision and Pattern Recognition.

[10] Lin T.Y., Dollár P., Girshick R., He K., Hariharan B., Belongie S. Feature Pyramid Networks for Object Detection. Proceedings of the 2017 IEEE Conference on Computer Vision and Pattern Recognition (CVPR).

[11] Huang G., Liu Z., Van Der Maaten L., Weinberger K.Q. Densely connected convolutional networks. Proceedings of the IEEE Conference on Computer Vision and Pattern Recognition.

[12] Chen Y., Li J., Xiao H., Jin X., Yan S., Feng J. (2017). Dual path networks. Adv. Neural Inf. Processing Syst..

[13] Girshick R. Fast r-cnn. Proceedings of the IEEE International Conference on Computer Vision.

[14] Dai J., Li Y., He K., Sun J. (2016). R-fcn: Object detection via region-based fully convolutional networks. Adv. Neural Inf. Processing Syst..

[15] Sermanet P., Eigen D., Zhang X., Mathieu M., Fergus R., LeCun Y. (2013). Overfeat: Integrated recognition, localization and detection using convolutional networks. arXiv.

[16] Redmon J., Divvala S., Girshick R., Farhadi A. You only look once: Unified, real-time object detection. Proceedings of the IEEE Conference on Computer Vision and Pattern Recognition.

[17] Jeong J., Park H., Kwak N. (2017). Enhancement of SSD by concatenating feature maps for object detection. arXiv.

[18] Zhou P., Ni B., Geng C., Hu J., Xu Y. Scale-transferrable object detection. Proceedings of the IEEE Conference on Computer Vision and Pattern Recognition.

[19] Fu C.-Y., Liu W., Ranga A., Tyagi A., Berg A.C. (2017). Dssd: Deconvolutional single shot detector. arXiv.

[20] Zhao Q., Sheng T., Wang Y., Tang Z., Chen Y., Cai L., Ling H. M2det: A single-shot object detector based on multi-level feature pyramid network. Proceedings of the AAAI Conference on Artificial Intelligence.

[21] Wu X., Sahoo D., Zhang D., Zhu J., Hoi S.C.H. (2020). Single-shot bidirectional pyramid networks for high-quality object detection. Neurocomputing.

[22] Liu S., Huang D., Wang Y. (2019). Learning spatial fusion for single-shot object detection. arXiv.

[23] Xie S., Girshick R., Dollár P., Tu Z., He K. Aggregated residual transformations for deep neural networks. Proceedings of the IEEE Conference on Computer Vision and Pattern Recognition.

[24] Lin T.Y., Goyal P., Girshick R., He K., Dollar P. (2020). Focal Loss for Dense Object Detection. IEEE Trans. Pattern Anal. Mach. Intell..

[25] Paszke A., Gross S., Massa F., Lerer A., Bradbury J., Chanan G., Killeen T., Lin Z., Gimelshein N., Antiga L. (2019). Pytorch: An imperative style, high-performance deep learning library. Adv. Neural Inf. Processing Syst..

[26] Everingham M., Van L., Christopher G., Williams K.I., Winn J., Zisserman A., Everingham M., Gool L.V., Williams C., Winn J. (2010). The Pascal Visual Object Classes (VOC) Challenge. Int. J. Comput. Vis..

[27] Lin T.-Y., Maire M., Belongie S.J., Hays J., Perona P., Ramanan D., Dollár P., Zitnick C.L. Microsoft coco: Common objects in context. Proceedings of the European Conference on Computer Vision.

[28] Oksuz K., Cam B.C., Kalkan S., Akbas E. (2020). Imbalance problems in object detection: A review. IEEE Trans. Pattern Anal. Mach. Intell..

[29] Liu L., Ouyang W., Wang X., Fieguth P., Chen J., Liu X., Pietikainen M. (2020). Deep Learning for Generic Object Detection: A Survey. Int. J. Comput. Vis..

